# American Dental Society of Europe

**Published:** 1894-11

**Authors:** Wm. S. Davenport


### NOTICES.

### American Dental Society of Europe.

The nineteenth annual meeting was held at Geneva, August 6th to 8th. The following officers were elected for the ensuing year:

President, Dr. Chas. W. Jenkins, Zurich.

Vice-President, Dr. Wm. Mitchell, London.

Treasurer, Dr. Chas. J. Monk, Wiesbaden.

Secretary, Dr. Wm. S. Davenport, Paris.

Executive Committee, Dr. Jenkins, Dr. G. C. Daboll and Dr. A. C. Hugenschmidt, of Paris.

Membership Committee, Dr. Mitchell, Dr. L. C. Bryan, of Basel, Dr. Waldo Royce, of Tunbridge Wells.

The next meeting will be held at Boulogne, the first Monday in August, 1895.

WM. S. DAVENPORT, *Secretary.*

